# Optical Waveguide Tunable Phase Delay Lines Based on the Superior Thermo-Optic Effect of Polymer

**DOI:** 10.3390/polym10050497

**Published:** 2018-05-04

**Authors:** Sung-Moon Kim, Tae-Hyun Park, Guanghao Huang, Min-Cheol Oh

**Affiliations:** Department of Electronics Engineering, Pusan National University, Pusan (Busan) 46241, Korea; sungmoon@pusan.ac.kr (S.-M.K.); taehyun@pusan.ac.kr (T.-H.P.); hgh@pusan.ac.kr (G.H.)

**Keywords:** polymer waveguide, phase delay line, thermo-optic effect, birefringence modulation

## Abstract

Strong thermo-optic effect of polymers is useful for fabricating tunable phase-delay devices with low power consumption and wide tuning range. However, if the temperature change is increased to extend the tuning range, an attenuation of the guided light is accompanied by the refractive index gradient across the waveguide core. For three different waveguide structures, in this study, the optimal structure of the device for a variable phase delay line was found by investigating the attenuation and interference phenomena accompanying phase modulation. By improving the uniformity of thermal distribution across the waveguide core, a phase delay of 130π rad was obtained with an output attenuation less than 0.5 dB.

## 1. Introduction

Tunable phase delay lines are useful devices for a variety of applications, such as optical sensors, optical communications, optical coherence tomography, LiDAR, photonic phased array antenna, and optical spectrometers [1,2,3,4,5,6]. Recently, research has been conducted to reduce the volume and cost, by constructing devices in the form of integrated optics using Si, SiN and polymer waveguides [7,8,9].

Integrated optical tunable phase delay lines control the phase of guided light passing through the waveguide in terms of the thermo-optic (TO) effect. The refractive index of polymer materials drops with heat application from a micro-heater formed around the waveguide [10]. However, when a large amount of heat is generated in the heater for large phase delay, undesired radiation of guided light results, causing output attenuation [11]. In addition, the radiated light causes interference with the guided light at the output port, thereby causing output power fluctuation, which deteriorates the accuracy of the sensor signal.

To prevent attenuation of the guided light even for large tuning, the index contrast between the core and the cladding needs to be increased, and the temperature uniformity has to be improved [12]. In this study, three types of tunable delay lines were designed and fabricated to reduce the attenuation and interference; the optimum structure was found by comparing the characteristics. In terms of the improved waveguide structure, maintaining an output attenuation at less than 0.5 dB, and a tunable delay of 0.33 ps/cm, was demonstrated for the first time in a polymeric optical waveguide device.

## 2. Device Design

**Figure 1:**
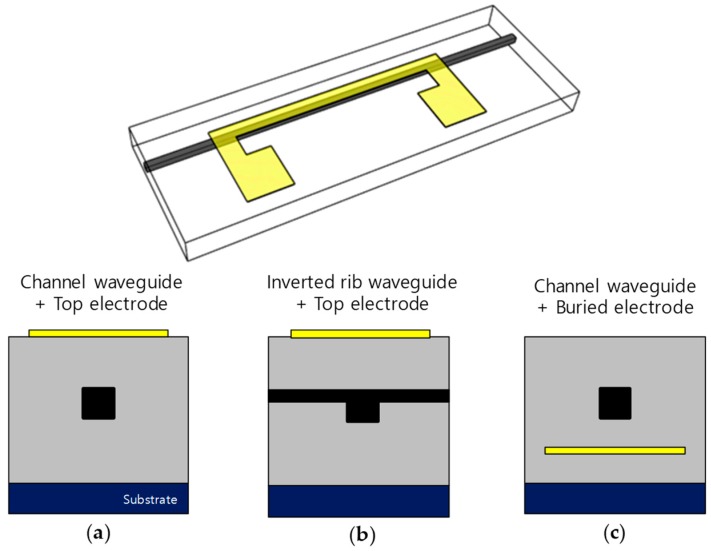
Waveguide structures of the phase delay line consisting of (**a**) channel waveguide with top electrode (type A), (**b**) inverted-rib waveguide with top electrode (type B), and (**c**) channel waveguide with buried bottom electrode (type C).

The tunable phase delay line has a simple structure consisting of a micro-heater made of a metal thin film placed near a straight optical waveguide. In order to compare the device characteristics, three types of tunable delay lines with different structures of the optical waveguides and heaters were fabricated, as shown in Figure 1. A conventional TO phase delay line (type A) has the structure of a buried channel waveguide with a heater on the top surface. The refractive indices of the core and cladding polymer materials are 1.4375 and 1.4300 respectively. According to the effective refractive index calculation, the single mode condition was satisfied for a waveguide with a width of 6 μm and a height of 5 μm. Heater width is 40 μm; the cross-section is shown in Figure 1a.

When the refractive index decreases due to the TO effect, radiation of the guided light occurs by the temperature induced refractive index gradient. To reduce this, one can increase the refractive index contrast between the optical waveguide core and the cladding material. Despite this large index contrast, to satisfy the single mode condition an inverted-rib waveguide structure (type B) is necessary, as shown in Figure 1b [13]. In this experiment, the refractive indices of the core and cladding materials were 1.455 and 1.440, respectively, and the refractive index contrast was 0.0150, which was twice as large as that of the type A. In the type C structure, the optical waveguide structure was the same as that of type A, while the heater was located at the bottom side for improving thermal distribution uniformity. Though the bottom electrode causes strong heat flow through the substrate to drop the power efficiency, the excellent TO effect of polymer could overcome this issue [14].

**Figure 2:**
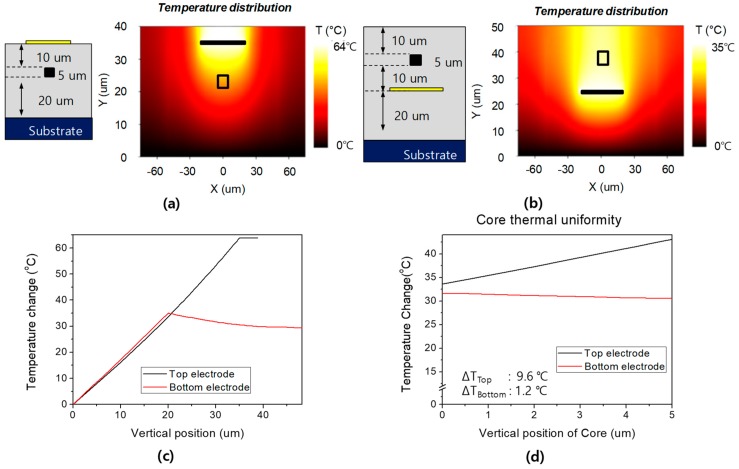
Temperature distributions on a waveguide cross-section for (**a**) top and (**b**) bottom electrode. Temperature changes for comparison in (**c**) vertical direction at *x* = 0, and (**d**) thermal uniformity across the core.

Temperature distribution induced by the heater was calculated using Optodesigner program of Phoenix Inc (Enschede, The Netherlands). Figure 2 shows 2D temperature distributions for the top and bottom heaters when 250 mW was applied to a 10 mm long heater. For the top electrode device, the temperature change at the heater was 63.8 °C, which was similar to 62.0 °C given by the formula in the reference [15]. The temperature change at *x* = 0 was compared for the top and bottom electrode cases, as in Figure 2c. In the bottom electrode, due to the heat flow to the substrate, the thermal heating efficiency was reduced, and the maximum temperature was lower than that of the top electrode. A buffer layer of 20 μm was used to improve the heat efficiency. The temperature uniformity inside the core was compared, as shown in Figure 2d, in which the temperature gradient was 9.6 °C for the top electrode structure, while it was reduced to 1.2 °C for the bottom electrode structure. Since the TO coefficient of the fluorinated polymer is −1.8 × 10^−4^/°C, the temperature change for a heater power of 250 mW is 30 °C, the refractive index change is −5.4 × 10^−3^, and the phase delay becomes 69.7π rad.

## 3. Fabrications of the Devices

**Figure 3:**
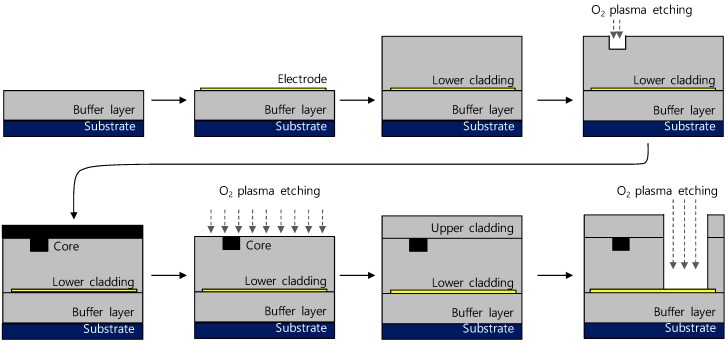
Fabrication procedure of the polymer waveguide phase delay line with a buried bottom electrode.

The tunable delay line device was fabricated using ZPU polymer materials produced by ChemOptics Inc (Daejeon, South Korea). The fabrication procedures for the bottom electrode structure (type C) is shown in Figure 3. ZPU polymers with refractive indexes of 1.4375 and 1.4300 were used as the core and cladding, respectively. The cladding ZPU polymer was firstly coated onto a silicon substrate to obtain a thickness of 20 μm as the heat insulating buffer layer, which was required only for the type C. For the heater electrode, Cr-Au was deposited at a thickness of 10–100 nm onto the substrate, and patterned using photo-lithography and wet etching. The lower cladding layer was coated to a thickness of 10 μm. A waveguide pattern was formed by photo-lithography process and etched by oxygen plasma equipment to make a channel with a width of 6 μm and a thickness of 5 μm. The core polymer was coated to fill up the channel, and then the entire surface was etched until the rectangular core shape was remained. The upper cladding ZPU polymer was coated with a thickness of 10 μm. Finally, a trench pattern was formed through plasma etching to expose the lower electrode. The length of the fabricated waveguide was 30 mm, and the electrode length was 10 mm. The resistance of the heater electrode was measured as 112, 92, and 144 Ω for the type A, B, and C structures, respectively.

## 4. Characterization

**Figure 4:**
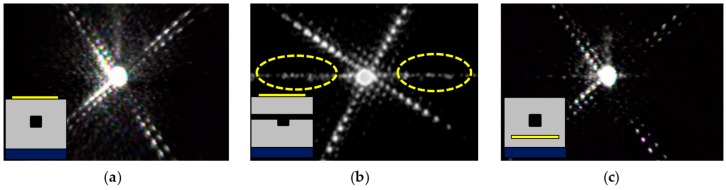
Waveguide output mode images for (**a**) channel waveguide with top electrode, (**b**) inverted rib waveguide with top electrode, and (**c**) channel waveguide with bottom electrode.

The fabricated devices were characterized using a distributed feedback (DFB) laser source with a wavelength of 1550 nm, and the input polarization state was set to TE in the waveguide using a fiber-optic polarization controller. As shown in the CCD images of Figure 4, the output modes of the channel waveguide of type A and C produces a well confined mode, while a radiated light confined in the lateral core layer was observed in the inverted rib waveguide of type B. This radiated light could interfere with the guided light and cause a fluctuation of the output signal during the heater operation for phase delay. Weak scattered light was also observed in Figure 4c, however, it was not strong enough to cause meaningful interference.

**Figure 5:**
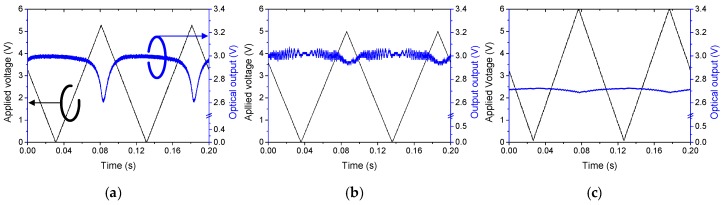
Output power variation accompanied by the TO phase modulation in the phase delay line consisting of (**a**) channel waveguide with top electrode, (**b**) inverted rib waveguide with top electrode, and (**c**) channel waveguide with bottom electrode.

The insertion loss measured with single mode fibers aligned to 3-cm long polymer waveguide device was about 1.6 dB, which could be divided into a propagation loss of 0.9 dB (0.3 dB/cm) and a coupling loss of 0.7 dB (0.35 dB/facet). To verify the output signal stability, a 10-Hz triangular wave voltage signal was applied on the heater with a maximum electrical power of 250 mW for each device. The type A device exhibited a significant attenuation with an additional loss of 14%, as shown in Figure 5a. Type B device prepared with an enhanced index contrast, the attenuation was reduced to 2.5%, as shown in Figure 5b. However, there was an output signal fluctuation with a short period that did not appear in type A. The signal fluctuation was due to the radiated light confined in the lateral core of inverted rib waveguide, as observed in Figure 4b. When the heater was operated, the phase of radiated light was also modulated: it then interferes with the guided light to produce the short period modulation signal. In the type C device shown in Figure 5c, the attenuation was the lowest at 1.1%, and no interference signal was observed.

**Figure 6:**
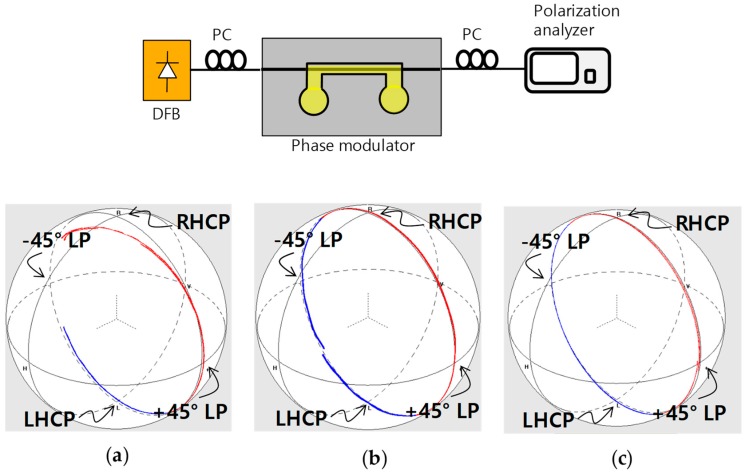
Birefringence modulation characteristics of (**a**) channel waveguide with top electrode (type A); (**b**) inverted rib waveguide with top electrode (type B), and (**c**) channel waveguide with bottom electrode (type C).

Throughout the experiment, we found that the TO phase delay line has a slight polarization dependence. This could be a useful effect for compensating initial polarization dependence of interferometric sensors, and for implementing an integrated-optic polarization controller [16,17]. For this purpose, a polarization modulation was demonstrated with a setup shown in Figure 6. A 45° linear polarization input was launched, and then output polarization state was monitored using a polarization analyzer. As shown in Figure 6, as the heater operated, the polarization state changed on the Poincaré sphere crossing the points of ±45° linear polarizations, left-handed circular polarization (LHCP), and right-handed circular polarization (RHCP). In type B and C devices, a complete circle in Poincaré sphere was obtained for the electrical power of 125 and 250 mW, respectively, as shown in Figure 6b,c, which corresponded to the 2π phase shift between the TE and TM modes. Birefringence modulation efficiency was higher in type B device because the rib waveguide possessed the higher initial birefringence, and the birefringence was relaxing as heat increased. In type A device, due to the attenuation, the circle was not closed, as shown in Figure 6a.

**Figure 7:**
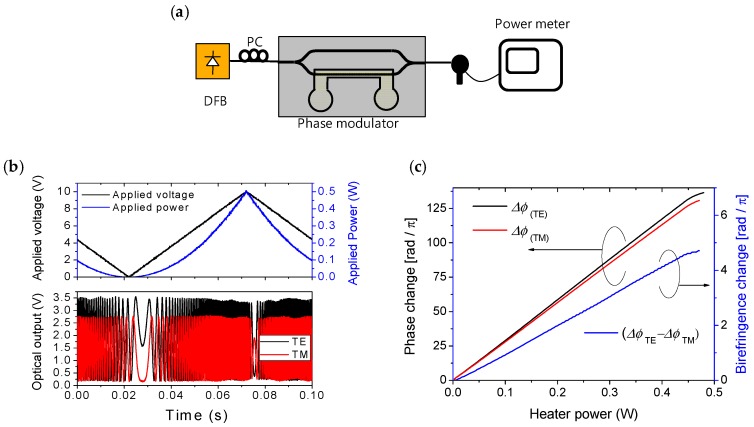
(**a**) Mach-Zehnder interferometer device for measuring the amount of TO phase change, (**b**) interference signals obtained for TE and TM polarizations, respectively, and (**c**) calculated phase change and the birefringence as a function of power dissipated in the heater.

In order to find an accurate amount of phase delay, a Mach-Zehnder (MZ) interferometer was fabricated in the type C structure. A fiber-optic polarization controller was used to define the input polarization to TE or TM, and then a heater on a path of the MZ was driven to produce an interference waveform as shown in Figure 7b. The amount of phase delay was calculated from the interference signal of Figure 7c. At 250 mW heating power, the phase delays of 73.5π and 70.6π rad were obtained for TE and TM respectively, and were found to be close to the designed value. Then, the heating power was increased to 480 mW until the attenuation was kept below 0.5 dB, and the phase delay was increased to 131π and 136π rad for TE and TM polarizations, respectively.

In this experiment, we produced a maximum phase delay efficiency of 13.6π rad/mm (0.033 ps/mm) in a polymer waveguide, which is almost same as that of silicon photonic devices [6]. However, in the silicon device, output power attenuation by 50% occurred due to the over-driving for maximum phase delay, which had to be compensated for later, by control circuits. In addition, the heater power for π phase shift, *P*_π_ for TE polarization was calculated to be 3.5 mW in the polymer device, which was much less than that of silicon photonic devices, *P*_π_ of 24.8 mW [7].

## 5. Conclusions

In this work, in order to optimize the polymer waveguide structure for the widely tunable phase delay, we fabricated three types of polymer waveguide devices with different waveguide structures and electrode positions, and compared the characteristics. Index contrast of the waveguide was enhanced, and the heater position was optimized for the purpose of reducing signal attenuation and spurious interferences that appear with phase delay. As a result, we demonstrated a tunable phase delay of up to 136π rad, with no significant attenuation. Fiber-to-fiber insertion loss was 1.6 dB. The phase delay efficiency per unit length was 13.6π rad/mm (0.033 ps/mm), which was comparable to that of the silicon device, while the power efficiency in polymer device was 7 times better.
